# Whole-Genome Shotgun Sequencing and Assembly of *Anopheles gambiae G3*, the Host of Malaria Parasite *Plasmodium sp*.

**DOI:** 10.7150/jgen.123168

**Published:** 2025-10-27

**Authors:** Eva English, Clara Gulick, Ashley Jane, Minseo Kim, Nat Kpodonu, Aden Lee, Ramon Kodi Suzuki Lopez, Krish Patel, Achyuta Rajaram, Leyla Unver, Anne E. Rankin, Shimaa M. Ghazal

**Affiliations:** 1 Science Department, Phillips Exeter Academy, Exeter, NH 03833, USA.

**Keywords:** *Anopheles gambiae* G3, Malaria, Next Generation Sequence, Illumina HiSeq, MASURCA, GenBank

## Abstract

*Anopheles gambiae* or the African malaria mosquito is the main vector of human malaria. G3*,* is an *Anopheles gambiae* strain, that was isolated from Gambiae in 1975, We report here a 216.3 Mbp draft genome sequence and assembly for *Anopheles gambiae* strain G3, with almost 82 thousand scaffold, 53X coverage and a G+C content of 44.5%.

## Introduction

Despite being considered eradicated from North America since 1951, Malaria remains one of the most fatal diseases in the world with over 240 million recorded cases per year according to the World Health Organization, About 2,000 cases of malaria are diagnosed in the United States annually, mostly in returned travelers, and an estimated of more than 600 thousand people died from Malaria around world in 2020 according to CDC 1. The disease is caused by a protozoon from the *Plasmodium* genus that is carried by the mosquito *Anopheles,* the human infection takes place during the blood meal of the female mosquito *Anopheles sp*.

*Anopheles* were first described in 1818 as a genus of mosquitoes. Many *anopheles* are vectors for *plasmodium* parasites which cause malaria in birds, reptiles, and mammals; however, *anopheles gambiae* is the only mosquito genus known to be a vector for human malaria transmitting *plasmodium falciparum.* 2

The clinical symptoms of malaria are largely a result of the replication of asexual states in human blood but transmission to mosquitoes is only achieved through the development of gametocytes in the bloodstream 3. Developed female mosquitos carrying malaria, which are exclusively capable of its transmission, are known to be more persistent in feeding attempts, feed on a more diverse selection of humans, feed more frequently 4,5,6 and suffer greater feeding-associated fatalities than uninfected females (7 and 8). An infected female mosquito first takes a blood meal. This process involves the mosquito numbing the site of the bite with its saliva. Saliva harbors sporozoites which infect humans. In the human the sporozoites proliferate in the liver forming a schizont which will eventually erupt and be exposed to the human blood stream. In the blood stream there are two outcomes. Firstly, the Erythrocytic cycle where the more infectious particles are formed; this stage is responsible for the clinical manifestations of the disease. The second outcome is the formation of gametocytes which exist in male and female forms. Once a second infected female mosquito has a blood meal, male and female gametocytes are joined to form a macrogametocyte through the sporogonic cycle. This process creates a oocyst in the mosquito which will burst forming more sporozoites allowing the mosquito to infect another human in the next blood meal. 1,7

Many species of *Anopheles* mosquitoes exist as part of species complexes; that is groups of very closely related species. *Anopheles gambiae* complex was recognized as a species complex in the 1960s, The individual species of the complex are morphologically difficult to distinguish from each other, although it is possible for larvae and adult females. The species exhibits different behavioral traits and includes the most important vectors of malaria in sub-Saharan Africa, particularly of the most dangerous malaria parasite.

## Materials and Methods

The *Anopheles gambiae*, strain G3 was isolated in 1975 in The Gambia, Africa. 1,2 DNA extraction was carried out from three adult female mosquitos that didn't have a blood meal, using the Quick-DNA Tissue/Insect Miniprep Kit (D6016, ZYMO Research, Irvine, California, USA) and following the manufacturer's instructions. This step immediately was followed by library preparation, using Illumina DNA Prep, (M) Tagmentation, Accession number: 20060060), and IDT® for Illumina® DNA/RNA UD Indexes Set A, Tagmentation (Accesion number: 20027213) were used. The library then sent to Hubbard Center for Genome Studies (University of New Hampshire, Durham, NH), where the draft genome of *Anopheles gambiae,* Strain G3 was generated using Illumina technology 9 techniques.

## Results

A standard Illumina shotgun library was constructed and sequenced using the Illumina HiSeq 2000 platform, which generated 216,250,770 bp. The Illumina sequence data were assembled using MaSuRCA v. 4.1.0 10, which combines the efficiency of the de Bruijn graph and Overlap-Layout-Consensus (OLC) approaches. The final draft assembly contained 83.491 contigs, with an N50 of 7.2 kb. The total size of the genome is 216.3 Mbp, and the final assembly is based on 3,341 Mb of Illumina draft data, which provided an average 53.0 × coverage of the genome, with a G+C content of 44.5%.

## Discussion

This paper is a report for draft genome sequence for *Anopheles gambiae* G3, and this whole-genome shotgun project has been deposited at DDBJ/EMBL/GenBank under the accession no. JAVFHU000000000.1. The version described in this paper is version JAVFHU000000000.1.

It is one of only nine genomes that were previously deposited in the Gene Bank for *Anopheles gambiae* G3 and one of four on the assembly level as scaffold, with 81,919 scaffolds for our strain. The list of all the nine genomes could be found here https://www.ncbi.nlm.nih.gov/datasets/genome/?taxon=7165. The data on the gene Bank showed the current gene set as 81.8% protein coding genes (12,518 genes), 8.8% non-coding genes (1,339 genes), 7% as small-RNAs (1,075 genes) and 2.4% pseudogenes (362 genes).
